# Supplementary material for: The interactome of intact mitochondria by cross-linking mass spectrometry provides evidence for coexisting respiratory supercomplexes
Source: Mol Cell Proteomics. 2017 Dec 8;17(2):216–32. doi: 10.1074/mcp.RA117.000470 (PMC5795388; doi:10.1074/mcp.RA117.000470)
Supplement: Supplemental Data [file supp_RA117.000470_133922_0_supp_23978_fzffwf.zip › spectra_annotation/mito_spectra_annotation/72-1-5-1-10-1.pdf]

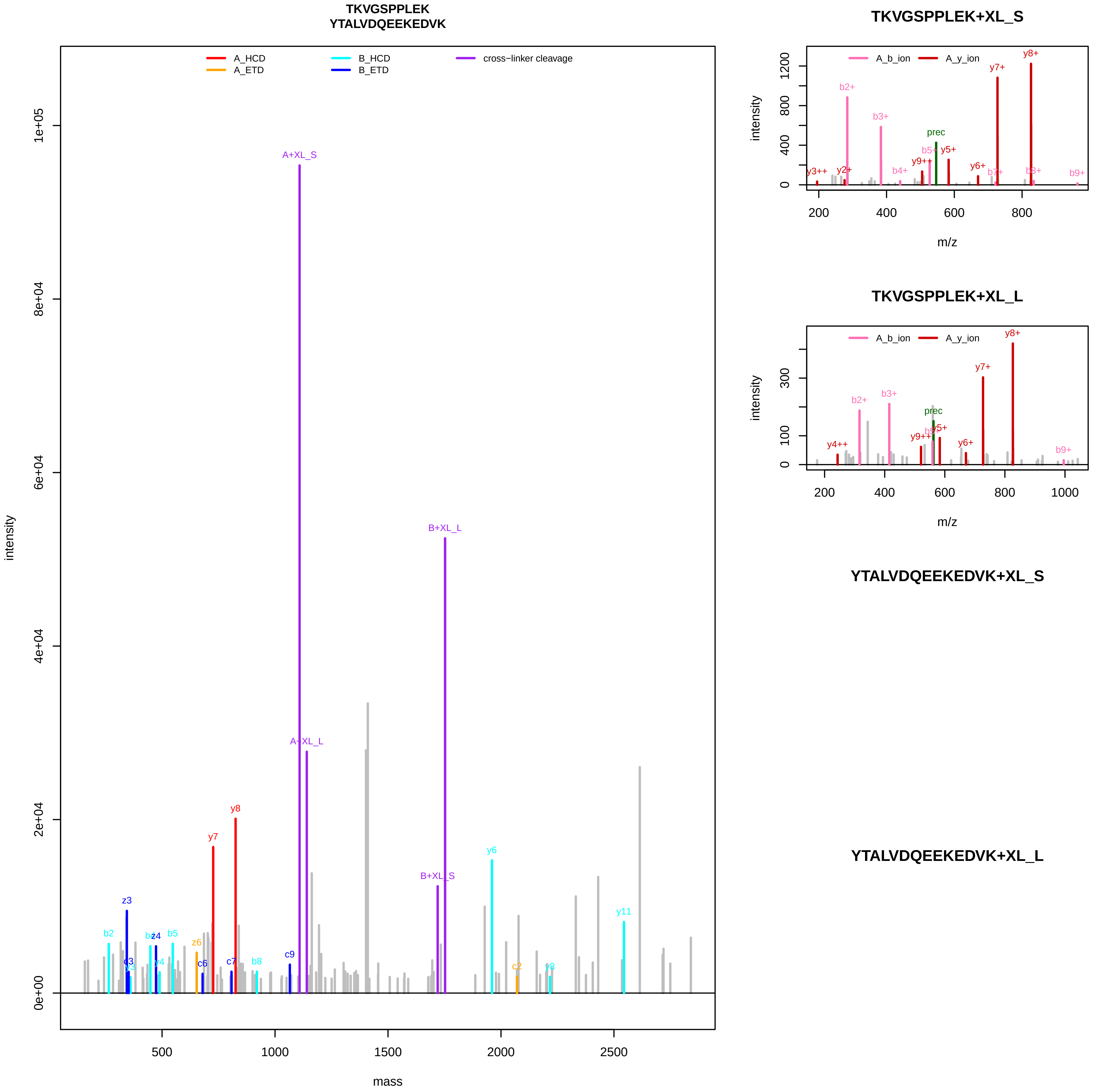
TKVGSPPLEK
YTALVDQEEKEDVK
A_HCD
A_ETD
B_HCD
B_ETD
cross−linker cleavage
A+XL_S
B+XL_L
A+XL_L
B+XL_S
y8
y7
y6
y11
z3
b2
c3
y3
b4
z4
y4
b5
z6
c6
c7
b8
c9
c2
y8
intensity
mass
0e+00
2e+04
4e+04
6e+04
8e+04
1e+05
500
1000
1500
2000
2500
TKVGSPPLEK+XL_S
A_b_ion
A_y_ion
y8+
y7+
b2+
b3+
prec
b5+
y5+
y9++
b4+
y6+
b7+
b8+
b9+
y3++
y2+
intensity
m/z
0
400
800
1200
200
400
600
800
TKVGSPPLEK+XL_L
A_b_ion
A_y_ion
y8+
y7+
b2+
b3+
prec
y5+
y9++
y6+
y4++
b9+
intensity
m/z
0
100
300
200
400
600
800
1000
YTALVDQEEKEDVK+XL_S
YTALVDQEEKEDVK+XL_L
